# *Pseudomonas aeruginosa* Induced Host Epithelial Cell Mitochondrial Dysfunction

**DOI:** 10.1038/s41598-019-47457-1

**Published:** 2019-08-15

**Authors:** Nicholas M. Maurice, Brahmchetna Bedi, Zhihong Yuan, Joanna B. Goldberg, Michael Koval, C. Michael Hart, Ruxana T. Sadikot

**Affiliations:** 1 Department of Medicine, Division of Pulmonary, Allergy, Critical Care, and Sleep Medicine, Emory University School of Medicine, Atlanta, GA 30322 USA; 2 Atlanta Veterans Affairs Health Care System, Decatur, GA 30033 USA; 3 Department of Pediatrics, Division of Pulmonology, Allergy/Immunology, Cystic Fibrosis, and Sleep, Emory University, Atlanta, GA 30322 USA; 4 Children’s Healthcare of Atlanta, Center for CF and Airways Disease Research Atlanta, Atlanta, GA USA; 5 Department of Cell Biology, Emory University School of Medicine, Atlanta, GA 30322 USA

**Keywords:** Molecular medicine, Mucosal immunology

## Abstract

The pathogenicity of *P*. *aeruginosa* is dependent on quorum sensing (QS), an inter-bacterial communication system that can also modulate host biology. The innate immune function of the lung mucosal barrier is dependent on proper mitochondrial function. The purpose of this study was to define the mechanism by which bacterial factors modulate host lung epithelial cell mitochondrial function and to investigate novel therapies that ameliorate this effect. 3-oxo-C12-HSL disrupts mitochondrial morphology, attenuates mitochondrial bioenergetics, and induces mitochondrial DNA oxidative injury. Mechanistically, we show that 3-oxo-C12-HSL attenuates expression of peroxisome proliferator-activated receptor-γ coactivator-1α (PGC-1α), a master regulator of mitochondrial biogenesis, antioxidant defense, and cellular respiration, and its downstream effectors in both BEAS-2B and primary lung epithelial cells. Overexpression of PGC-1α attenuates the inhibition in cellular respiration caused by 3-oxo-C12-HSL. Pharmacologic activation of PGC-1α restores barrier integrity in cells treated with 3-oxo-C12-HSL. These data demonstrate that the *P*. *aeruginosa* QS molecule, 3-oxo-C12-HSL, alters mitochondrial pathways critical for lung mucosal immunity. Genetic and pharmacologic strategies that activate the PGC-1α pathway enhance host epithelial cell mitochondrial function and improve the epithelial innate response to *P*. *aeruginosa*. Therapies that rescue PGC-1α function may provide a complementary approach in the treatment of *P*. *aeruginosa* infection.

## Introduction

*Pseudomonas aeruginosa* is a ubiquitous gram-negative bacterium responsible for a variety of opportunistic infections in humans. *P*. *aeruginosa* is an especially important respiratory tract pathogen causing acute infections in susceptible patients such as those who are hospitalized, critically ill, ventilated, or immune-compromised. *P*. *aeruginosa* is also responsible for chronic recalcitrant infections in patients with cystic fibrosis (CF) and non-CF bronchiectasis. These infections are associated with significant morbidity and mortality in vulnerable patient populations^1^. Furthermore, the emergence and spread of drug resistance among *P*. *aeruginosa* strains is a growing health threat that calls for the development of novel strategies that not only kill or inhibit the growth of bacteria, but also target bacterial virulence mechanisms or alternatively enhance the host immune response to infection^2^.

The pathogenicity of *P*. *aeruginosa* depends in large part on its genetic flexibility made possible by a complex genome and arsenal of virulence factors. QS is a central virulence factor that allows *P*. *aeruginosa* to coordinate expression of genes important to adaptation to the environment. This mechanism enables the bacteria to regulate genes in a density-dependent manner through the production of acyl homoserine lactones, small diffusible molecules that act as auto-inducers^3^. The primary QS molecule is N-(3-Oxododecanoyl)-L-homoserine lactone (3-oxo-C12-HSL)^4^. QS mediates *P*. *aeruginosa* survival, virulence, and biofilm formation. Strains that lack QS capacity demonstrate reduced pathogenicity^5^. QS molecules are also clinically relevant to human disease. QS molecules accumulate during the growth of *P*. *aeruginosa in vitro* reaching maximal concentrations at the end of the exponential phase and then remaining stable for at least 24 hours^6^. QS molecules can be isolated from lung tissue and the sputum of CF patients with chronic *P*. *aeruginosa* infections^7^. In addition, the detection of QS molecules in tracheal aspirates of intubated patients predicts the conversion from colonization to *P*. *aeruginosa* ventilator-associated pneumonia^8^.

The host response to *P*. *aeruginosa* is complex and involves the coordinated activity of multiple cell types. Notably, the lung epithelium constitutes the first line of defense against *P*. *aeruginosa*, providing a physical barrier to microbial invasion through a network of cell-cell contacts including tight junctions. The mucociliary clearance in the upper respiratory tract prevents the establishment of *P*. *aeruginosa* infection. Additionally, epithelial cells recognize *P*. *aeruginosa* by various receptors and activate signal transduction pathways that result in production of inflammatory cytokines and chemokines that recruit innate and adaptive immune cells^9^.

Mitochondrial metabolic activity is necessary for normal cellular function in the lung. Due to their critical bioenergetic function of producing ATP through the process of oxidative phosphorylation (OXPHOS), mitochondria are critical for many functions of lung epithelial cells including the maintenance of barrier integrity, ciliary function, and fluid balance^10,11^. Abnormal bioenergetics is increasingly recognized as central to many pulmonary pathologic conditions^12,13^.

A byproduct of OXPHOS is the generation of reactive oxygen species (ROS), but these are normally tightly regulated by antioxidant systems. However, cellular stresses that increase demand for ATP or disrupt the OXPHOS pathway can cause increased ROS generation and overwhelm antioxidant defenses. This can lead to further disruption of mitochondrial metabolic processes, endoplasmic reticulum Ca^2+^ release, cell death, damage of mitochondrial DNA (mtDNA), and promotion of inflammatory signaling^13^.

Mitochondrial biogenesis, the process of growth and division of pre-existing mitochondria, allows cells to quickly replace damaged mitochondria. This quality control mechanism is closely linked with mitophagy, the process by which damaged mitochondria are selectively removed through lysosomal degradation. Mitochondrial biogenesis is activated within type 2 alveolar epithelial cells during the recovery phase of a murine model of treated *Staphylococcus aureus* pneumonia^14^. In chronic smoking-related lung disease and pulmonary fibrosis, evidence suggests that dysregulation of mitochondrial biogenesis pathways within the lung epithelium may be central to the pathogenesis of these disorders^15,16^. However, there is a scarcity of information regarding the effect of *P*. *aeruginosa* on the regulation of mitochondrial biogenesis in the lung epithelium.

PGC-1α is a transcriptional coactivator that acts as a master regulator of mitochondrial biogenesis, mitochondrial respiration, and antioxidant activity. PGC-1α interacts with nuclear and mitochondrial transcription factors including PPARγ, nuclear respiratory factors 1 and 2 (NRF1, NRF2), estrogen related receptor α (ERRα), and mitochondrial transcription factor-A (TFAM). PGC-1α is both transcriptionally regulated and also activated by post-translational modifications including deacetylation by Sirtuin (SIRT) 1, an NAD^+^-dependent deacetylase, and phosphorylation by AMP-dependent kinase (AMPK). Since AMPK and SIRT1 activity are regulated by the cellular energy supply (AMP/ATP ratio and NAD^+^ levels, respectively), PGC-1α activation is stimulated by the depletion of bioenergetic capacity^17^.

In this study, we examined the effects of the *P*. *aeruginosa* QS molecule, 3-oxo-C12-HSL on the mitochondrial function of bronchial epithelial cells and investigated the hypothesis that activation of the PGC-1α pathway could ameliorate these effects. We demonstrate that the QS molecule disrupts mitochondrial structure, attenuates cellular respiration, induces ROS generation, and activates the apoptosis pathway within bronchial epithelial cells. Most importantly, overexpression of PGC-1α partially ameliorates the attenuation of mitochondrial respiration. Additionally, pharmacologic activation of PGC-1α with metformin or resveratrol restores barrier integrity following exposure to the QS molecule. These studies provide novel mechanistic insight into the role of PGC-1α in the epithelial host response to *P*. *aeruginosa* infection.

## Results

### *P. aeruginosa* QS molecules disrupt mitochondrial morphology in bronchial epithelial cells

**Figure 1:**
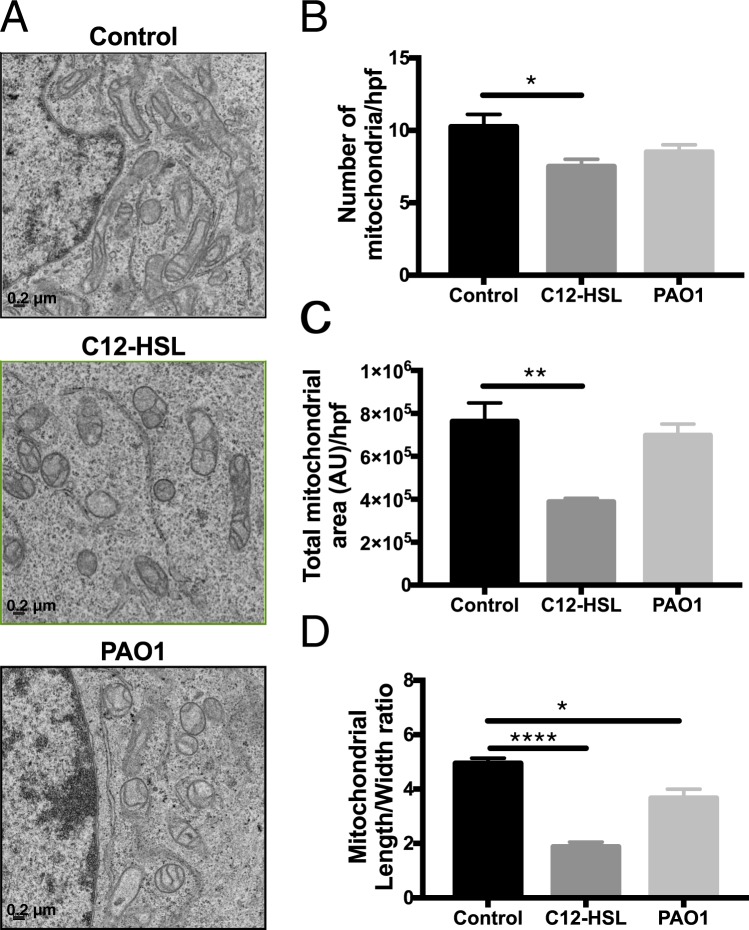
*P*. *aeruginosa* QS molecules disrupt mitochondrial morphology in bronchial epithelial cells. BEAS-2B cells were treated with vehicle control (DMSO), 100 μM 3-oxo-C12-HSL (C12-HSL), or infected with PAO1 (MOI 20) for 6 hours. Cells were then fixed and processed for electron microscopy (EM) analysis. (**A**) representative high-powered field (hpf) images of treatment groups, 15,000x magnification, scale 0.2 μm. (**B**) number of mitochondria per hpf. (**C**) Total mitochondrial area per hpf. (**D**) Mitochondrial length to width ratio. 3-oxo-C12-HSL and PAO1 disrupt mitochondrial morphologic parameters. Results are mean ± SEM. *P < 0.05, **p < 0.01, ****p < 0.0001 all vs. control. One-way ANOVA with Tukey’s multiple comparisons test used for statistical analysis.

Mitochondria, which are central to cell metabolism and also important mediators of other cellular pathways including those that regulate cell death, are frequent targets of microbial products^18^. Mitochondrial morphology is tightly regulated by dynamic mitochondrial quality control mechanisms including mitochondrial biogenesis, mitophagy, fusion, and fission. Changes in mitochondrial morphology can be associated with perturbations in mitochondrial function^19^. Previous research has demonstrated that the QS molecule, 3-oxo-C12-HSL, activates the apoptotic pathway in a variety of cell types^20–22^ and causes mitochondrial membrane permeabilization in fibroblasts^20^, but little is known about their specific effects on lung epithelial cells. We hypothesized that QS molecules would disrupt mitochondrial morphology in bronchial epithelial cells. Indeed, a 6-hour treatment with 100 μM 3-oxo-C12-HSL had a profound qualitative effect on mitochondrial morphology and cristae structure (Fig. 1A). Quantitatively, it caused significant decrease in number of mitochondrial per high power field (Fig. 1B), mitochondrial area (Fig. 1C), and length-to-width ratio (Fig. 1D) as detected by electron microscopic morphometric analysis. Infection with *P*. *aeruginosa* strain, PAO1 (MOI 20) for 6 hours had a similar effect on mitochondrial length to width ratio (Fig. 1D). Collectively, these data demonstrate that *P*. *aeruginosa* and its QS molecules may evade host defenses by disrupting host epithelial mitochondria.

### 3-oxo-C12-HSL attenuates metabolic potential and mitochondrial respiration in bronchial epithelial cells

**Figure 2:**
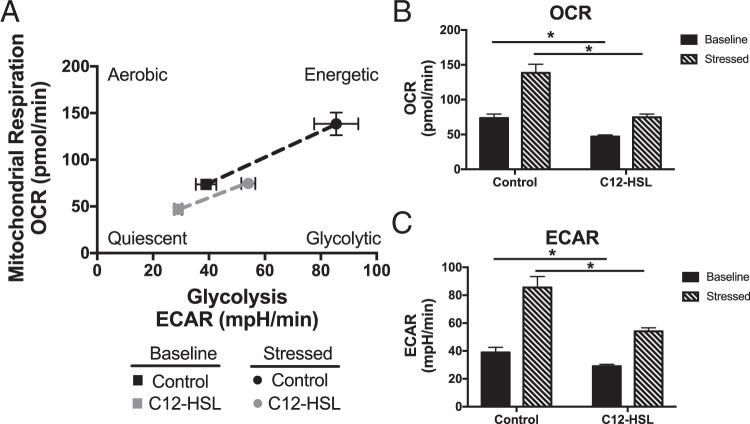
*P*. *aeruginosa* QS molecules disrupt bronchial epithelial cell bioenergetics and metabolic potential. BEAS-2B cells were treated with vehicle control (DMSO) or 100 μM 3-oxo-C12-HSL for 6 hours. Cells were then analyzed using Seahorse Cell Energy Phenotype assay. This allows for the real time measurement of oxygen consumption rate (OCR) and extracellular acidification rate (ECAR), which are representative of mitochondrial respiration and glycolysis respectively, at rest and after induction of a bioenergetic stress caused by treatment with the ATP synthase inhibitor, oligomycin, and the uncoupling agent, FCCP. (**A**) Plot of baseline and stressed conditions in control and 3-oxo-C12-HSL-treated cells. 3-oxo-C12-HSL-treated cells are more quiescent at baseline and have less metabolic potential under stressed conditions. (**B**) OCR measurements at baseline and stressed conditions. 3-oxo-C12-HSL decreases basal and stressed OCR as compared to control. (**C**) ECAR measurements at baseline and stressed conditions. 3-oxo-C12-HSL decreases basal and stressed ECAR as compared to control. Results are mean ± SEM. *p < 0.05, n = 3 independent experiments. One-way ANOVA with Tukey’s multiple comparisons test used for statistical analysis.

Since at least 90% of cellular energy is produced by mitochondria^23^, we wanted to investigate whether the changes in mitochondrial structure reflect a disruption in cellular bioenergetics. We utilized the Seahorse XF Cell Energy Phenotype test, which allows for the real time measurement of both extracellular acidification rate (ECAR), a measure of glycolysis, and oxygen consumption rate (OCR), a measure of oxidative phosphorylation. Because the assay did not provide reliable data in cells treated with live bacteria because of microbial metabolism that interfered with the assay, we focused on the specific effect of QS molecules on cellular bioenergetics (Fig. 2A). A 6-hour treatment with 100 μM 3-oxo-C12-HSL resulted in a significant decrease in baseline OCR (Fig. 2B) and ECAR (Fig. 2C) levels. In addition, cells treated with the QS molecule, had less metabolic potential to increase the rates of glycolysis and OXPHOS in response to the stress caused by the addition of an uncoupling agent, FCCP, and the ATP synthase inhibitor, oligomycin, (Fig. 2B,C).

**Figure 3:**
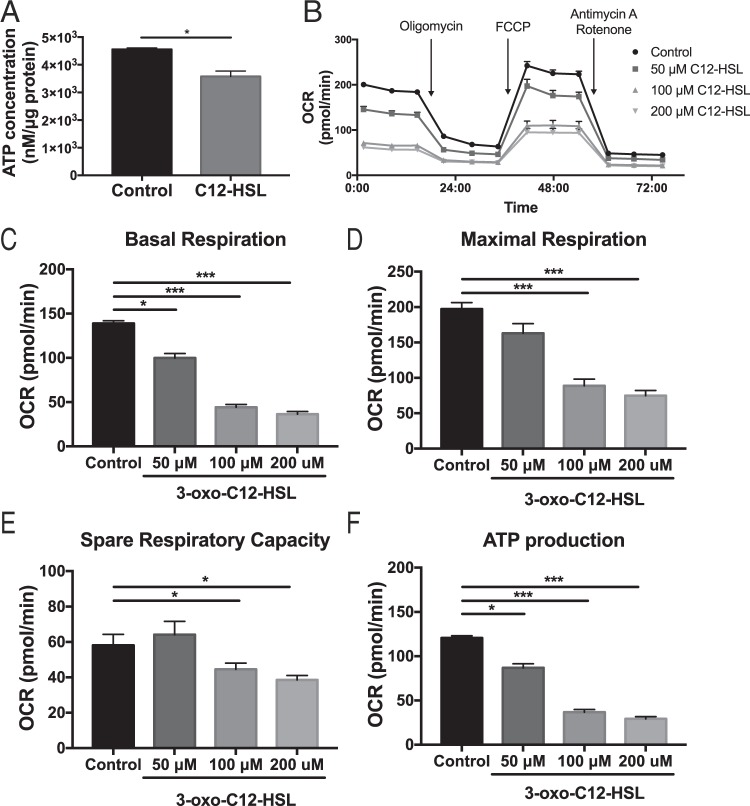
3-oxo-C12-HSL attenuates mitochondrial respiration in bronchial epithelial cells. (**A**) BEAS-2B cells were treated with 100 μM 3-oxo-C12-HSL for 6 hours and then the amount of ATP normalized to protein was measured in cellular lysates. 3-oxo-C12-HSL significantly reduced the level of ATP present in cells. (**B**–**F**) BEAS-2B cells were treated with 50, 100, or 200 μM 3-oxo-C12-HSL for 6 hours and then analyzed using the Seahorse XF Mito Stress Test assay. (**B**) OCR was measured real time at baseline and then in response to a series of injections to interrogate various aspects of the electron transport chain. 3-oxo-C12-HSL significantly attenuated basal respiration (**C**) maximal respiration (**D**) spare respiratory capacity (**E**) and ATP-linked respiration (**F**). Results are mean ± SEM. For *A*, * p < 0.05 by unpaired two-tailed t test, n = 3 independent experiments. For *C-F* * p < 0.05, **p < 0.01, ***p < 0.001 by one-way ANOVA with Tukey’s multiple comparisons test, n = 6 independent experiments.

Next, we wanted to specifically interrogate the effect of the QS molecule on various aspects of the OXPHOS pathway. First, we analyzed whether 3-oxo-C12-HSL reduced steady-state levels of ATP, since OXPHOS is the primary cellular process by which ATP is generated. In cells treated with 100 μM 3-oxo-C12-HSL for 6-hours, there was a significant decrease in ATP concentration normalized to intracellular protein (Fig. 3A). Next, we employed the Seahorse XF Mito Stress Test to interrogate various aspects of the mitochondrial respiration pathway (Fig. 3B). We found that a 6-hour treatment with 50–200 μM 3-oxo-C12-HSL attenuated basal respiration (Fig. 3C), maximal respiration (Fig. 3D), spare respiratory capacity (Fig. 3E), and ATP production (Fig. 3F) in a dose-dependent manner. Together, these data suggest that QS molecules compromise mitochondrial respiration in epithelial cells, and this effect may result in reduced immune function.

### 3-oxo-C12-HSL decreases mitochondrial content in bronchial epithelial cells

**Figure 4:**
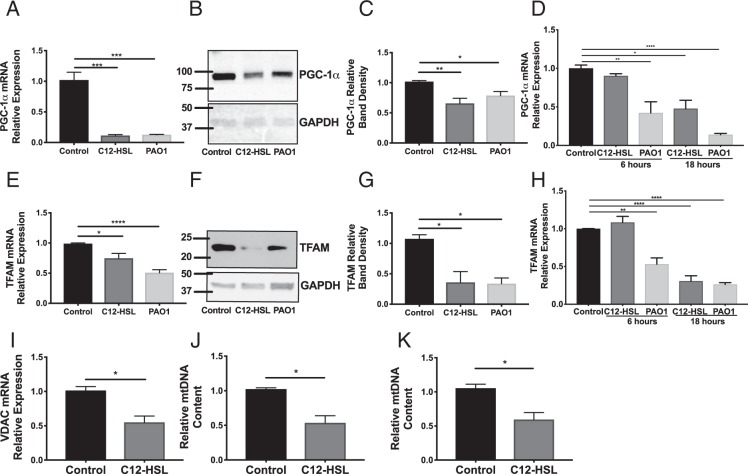
*P*. *aeruginosa* infection and the bacterial QS molecule attenuates expression of PGC-1α and TFAM and reduces mitochondrial biogenesis in bronchial epithelial cells. (**A**–**C**,**E**–**G**,**I**–**J**) BEAS-2B cells were treated with 100 μM 3-oxo-C12-HSL or infected with PAO1 (MOI 1) for 16 hours. Relative mRNA (**A**,**E**,**I**) and protein expression (**B**,**F** *f*or representative western blots, (**C**,**G** for *normalized densitometry*) of PGC-1α (**B**,**C**) and TFAM (**F**,**G**) were measured. The full-length blots are presented in Supplementary Figs 1 and 2. Both PAO1 and 3-oxo-C12-HSL significantly attenuated expression of PGC-1α and TFAM in BEAS-2B cells. (**D**,**H**,**K**) Human primary bronchial epithelial cells (NhBEs) grown at an air-liquid interface were also treated with 100 μM 3-oxo-C12-HSL or infected with PAO1 (MOI 1) for 6 and 18 hours and relative mRNA expression for PGC-1α (**D**) and TFAM (**H**) were measured. PAO1 attenuated expression of PGC-1α and TFAM at both 6 and 18 hours. 3-oxo-C12-HSL attenuated expression of PGC-1α and TFAM at 18 hours. (**I**) In BEAS-2B cells, 3-oxo-C12-HSL reduced relative mRNA expression of the mitochondrial marker VDAC1. In BEAS-2B cells (**J**) and NhBEs (**K**) 3-oxo-C12-HSL reduced relative mtDNA content normalized to nuclear DNA. Results are mean ± SEM. * p < 0.05, **p < 0.01, ***p < 0.001, ****p < 0.0001 by one-way ANOVA with Tukey’s multiple comparisons test, n = 6–8 independent experiments (**A**,**C**,**E**,**G**,**I**) n = *3* independent experiments (**D**,**H**). For (**I**,**J**,**K**) results are mean ± SEM.*p < 0.05 by unpaired t test, n = 3 independent experiments.

Others have demonstrated that cellular bioenergetic crises are generally a potent stimulus for activation of the mitochondrial biogenesis pathway in order to create new mitochondria to replace those that have been damaged^24^. We hypothesized that the QS molecule would similarly cause an increase in mitochondrial biogenesis in host cells. Mitochondrial biogenesis is principally regulated by PGC-1α, a transcriptional co-activator that promotes the transcription of nuclear and mitochondrial genes crucial for mitochondrial function^25,26^. Interestingly, 16-hour treatment with 100 μM 3-oxo-C12-HSL or PAO1 (MOI 1) resulted in a significant decrease in the mRNA and protein levels of PGC-1α (Fig. 4A–C, Supplementary Fig. 1) and its downstream effector, TFAM (Fig. 4E–G, Supplementary Fig. 2) in BEAS-2B cells. In primary human bronchial epithelial cells, 100 μM 3-oxo-C12-HSL and infection with PAO1 similarly reduced expression of PGC-1α and TFAM (Fig. 4D,H). 3-oxo-C12-HSL also caused a significant decrease in expression of the mitochondrial specific protein, voltage-dependent anion-selective channel (VDAC) 1 (Fig. 4I). Furthermore, it significantly decreased relative mtDNA content normalized for nuclear DNA in BEAS-2B cells (Fig. 4J) and in human primary bronchial epithelial cells (NhBEs) (Fig. 4K). These findings together confirm that 3-oxo-C12-HSL not only attenuates mitochondrial bioenergetics but also suppresses the ability of the cell to regenerate new mitochondria through biogenesis to respond to this energetic stress.

### 3-oxo-C12-HSL induces ROS generation, oxidative mitochondrial DNA damage, and the apoptotic pathway in bronchial epithelial cells

**Figure 5:**
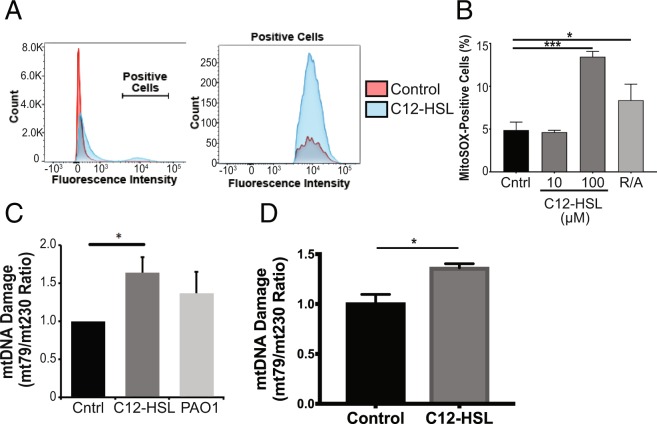
*P*. *aeruginosa* QS molecule induces ROS generation and oxidative mtDNA injury in bronchial epithelial cells. (**A**–**C**) BEAS-2B cells were treated with 3-oxo-C12-HSL (10 μM, 100 μM), or PAO1 (MOI 20), or a positive control (rotenone and antimycin A, R/A) for 6 hours. MitoSOX fluorescence was measured using flow cytometry. (**A**) Mean fluorescence intensity is displayed in a histogram format. Cells that stained positive for MitoSOX fluorescence (labeled Positive Cells) were quantified and compared between groups (**B**). 100 μM 3-oxo-C12-HSL resulted in a significant increase in ROS generation. (**C**,**D**) The ratio of a 79 bp mtDNA fragment to 230 bp mtDNA fragment was used to quantify mtDNA oxidative damage in BEAS-2B cells (**C**) and primary NhBEs (**D**) treated with 100 μM 3-oxo-C12-HSL or PAO1 for 6 hours. 100 μM 3-oxo-C12-HSL produced significant mtDNA oxidative injury in both BEAS-2B and NhBEs. *A-C*, Results are mean ± SEM. *p < 0.05, ***p < 0.001 by one-way ANOVA with Tukey’s multiple comparisons test. n = 3 independent experiments. (**D**) Results are mean ± SEM. *p < 0.05, unpaired t test, n = 3 independent experiments

Electron transport chain complexes regularly leak a small percentage of electrons to partially reduce oxygen to produce superoxide anion, which can go on to produce additional ROS. Because of the potential damage that can be caused by these ROS, there are several antioxidant defenses within mitochondria. The balance between ROS and antioxidant defenses is tightly regulated under physiologic conditions, but under pathologic conditions, this balance can be disrupted leading to excessive ROS generation, oxidative injury, and apoptosis^27^. We utilized MitoSOX™ Red, a fluorogenic dye, which specifically detects superoxide anion, to determine the effect of the QS molecule, 3-oxo-C12-HSL on ROS generation. Flow cytometry analysis demonstrated a noticeable increase in fluorescence intensity in cells treated with 3-oxo-C12-HSL (Fig. 5A). 3-oxo-C12-HSL caused a significant increase in cells staining positive for MitoSOX™ as compared to control and on the order with the positive control, the respiratory chain toxins, rotenone and antimycin A (Fig. 5B).

Since mtDNA is particularly susceptible to ROS generation, we next investigated whether the QS molecule enhances mtDNA damage. Indeed, 3-oxo-C12-HSL caused a significant increase in mtDNA damage as measured by an increase in the mt79/mt230 DNA fragment ratio (Fig. 5C) in BEAS-2B cells. This effect was also seen in human primary lung epithelial cells treated with 3-oxo-C12-HSL (Fig. 5D).

**Figure 6:**
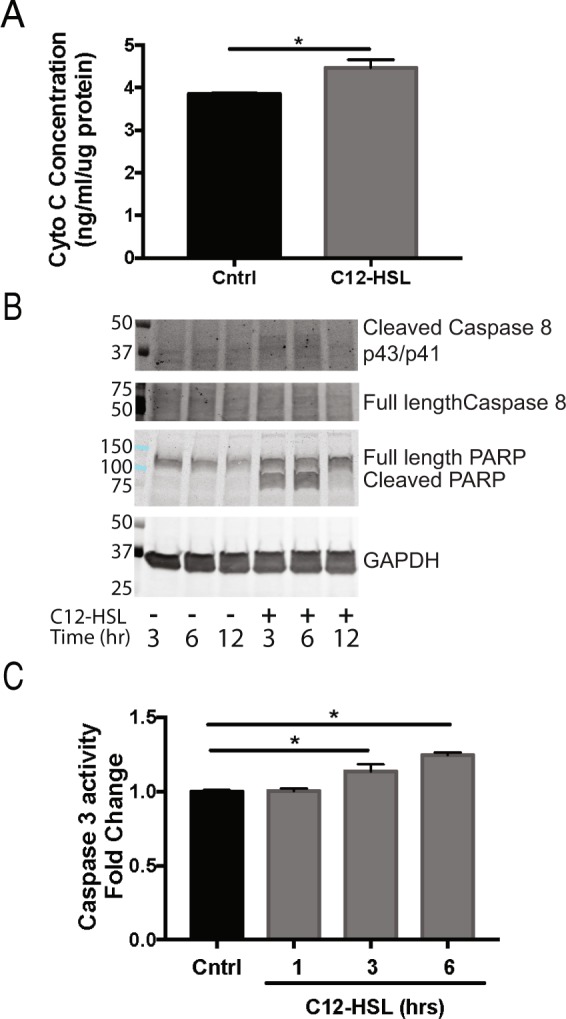
*P*. *aeruginosa* QS molecule triggers the apoptosis pathway. (**A**) BEAS-2B cells were treated with 100 μM 3-oxo-C12-HSL for 6 hours. The intracellular quantity of cytochrome c normalized to protein was quantified in the two groups. (**B**) BEAS-2B cells were treated with 100 μM 3-oxo-C12-HSL for 3, 6, and 12 hours and then protein was isolated and used for immunoblotting for the cleaved and full-length forms caspase-8 and PARP relative to the endogenous control, GAPDH. Immunoblots shown are representative of three independent experiments. (**C**) BEAS-2B cells were treated with100 μM 3-oxo-C12-HSL for 1, 3, and 6 hours and caspase-3 activity was measured. 3-oxo-C12-HSL induced cytochrome c release, caspase-8 cleavage, caspase-3 activation, and PARP cleavage. (**A**,**C**) Results are mean ± SEM. *p < 0.05 by unpaired t test (**A**) or one-way ANOVA with Tukey’s multiple comparisons test (**C**) n = 3 independent experiments.

Finally, because excessive ROS are a known stimulus for induction of cell death, we interrogated whether the apoptosis pathway was activated in response to the mitochondrial damage and oxidative stress caused by 3-oxo-C12-HSL. The QS molecule caused a significant increase in cytochrome C release (Fig. 6A), an early event in the mitochondrial-dependent intrinsic apoptosis pathway, but activation of the intrinsic pathway initiator caspase, caspase-9, was not observed (Supplementary Fig. 3). Instead, cleavage of the extrinsic pathway initiator caspase, caspase-8, was induced by 3-oxo-C12-HSL at 3 and 6 hours (Fig. 6B, Supplementary Fig. 3). The QS molecule also activated the executioner caspase, caspase-3 (Fig. 6C) and downstream cleavage of Poly (ADP-ribose) Polymerase (PARP) (Fig. 6B, Supplementary Fig. 3). Collectively, these data show that *P*. *aeruginosa* QS molecules induce production of mitochondrial ROS with oxidative mtDNA damage and triggers the apoptotic pathway. Since epithelial cells are critical for host defenses for *P*. *aeruginosa*, these damaging effects likely contribute to impaired epithelial immune response.

### Overexpression of PGC-1α partially rescues 3-oxo-C12-HSL-induced impairment in mitochondrial biogenesis and respiration

PGC-1α is a master regulator of cellular respiration, mitochondrial biogenesis, and anti-oxidant defenses. Given its central role in the pathways that are perturbed by the QS molecule, 3-oxo-C12-HSL, we hypothesized that overexpression of PGC-1α would ameliorate some of its deleterious effects.

**Figure 7:**
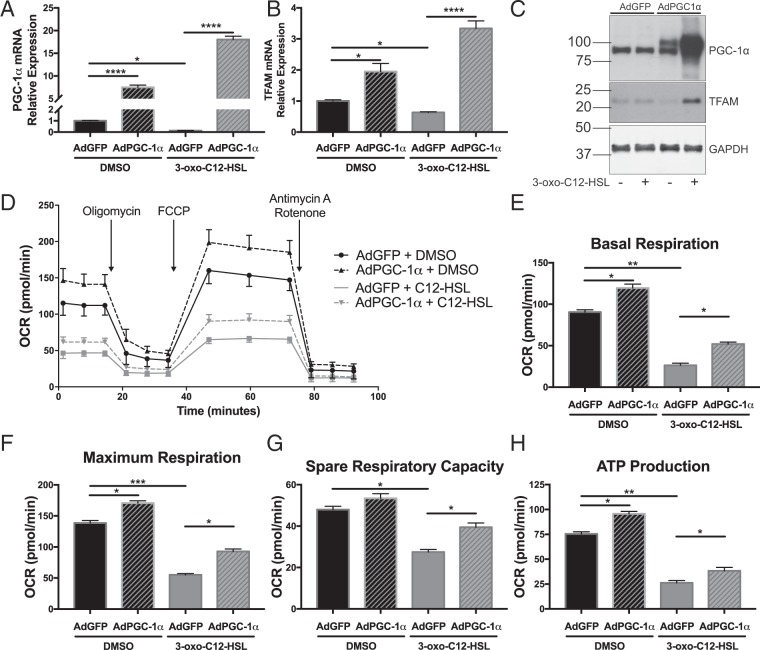
Overexpression of PGC-1α partially rescues 3-oxo-C12-HSL-induced impairment in mitochondrial biogenesis and cellular respiration. BEAS-2B cells were transduced with adenovirus (MOI 25) expressing GFP (AdGFP) or PGC-1α (AdPGC-1α) for 48 hours (**A**,**B**) or 72 hours (**C**–**H**) prior to treatment with 100 μM 3-oxo-C12-HSL. QPCR for PGC-1α (**A**) and TFAM (**B**) and western blot representative of three independent experiments for PGC-1α and TFAM (**C**) confirms that PGC-1α overexpression prevents the attenuation in the expression of PGC-1α and TFAM caused by 3-oxo-C12-HSL. Full length blots are presented in Supplementary Fig. 3D–H. Seahorse XF Mito Stress Test analysis demonstrates that in cells treated with 3-oxo-C12-HSL, PGC-1α overexpression significantly increased basal respiration (**E**) maximal respiration (**F**) spare respiratory capacity, and ATP-linked respiration (**G**). Results are mean ± SEM. *p < 0.05, **p < 0.01, ***p < 0.001, ****p < 0.0001 by one-way ANOVA with Tukey’s multiple comparisons test, n = 4 (**A**–**C**) or 6 (**D**–**G**) independent experiments.

BEAS-2B cells were either untransduced or transduced with adenovirus (MOI 25) expressing either GFP or PGC-1α. AdGFP transduction had no significant effect on gene expression compared to untransduced cells (data not shown). PGC-1α mRNA and protein overexpression was achieved with AdPGC-1α transduction at 48 and 72 hours, respectively (Fig. 7A,C, Supplementary Fig. 4). In addition, TFAM levels were restored with AdPGC-1α transduction (Fig. 7B,C, Supplementary Fig. 4).

Using the SeahorseXF Mito Stress Test assay, we also found that overexpression of PGC-1α partially restored parameters of mitochondrial respiration in cells treated with the QS molecule (Fig. 7D–H). In particular, there was a significant increase in basal respiration (Fig. 7E), maximum respiration (Fig. 7F), spare respiratory capacity (Fig. 7G), and ATP production (Fig. 7H), in 3-oxo-C12-HSL-treated cells that were overexpressing PGC-1α as compared with AdGFP controls. Together, these data show that overexpression of PGC-1α can at least partially rescue mitochondrial biogenesis pathways and cellular respiration that is impaired by the QS molecules.

### Metformin and resveratrol restore epithelial barrier integrity following 3-oxo-C12-HSL treatment

**Figure 8:**
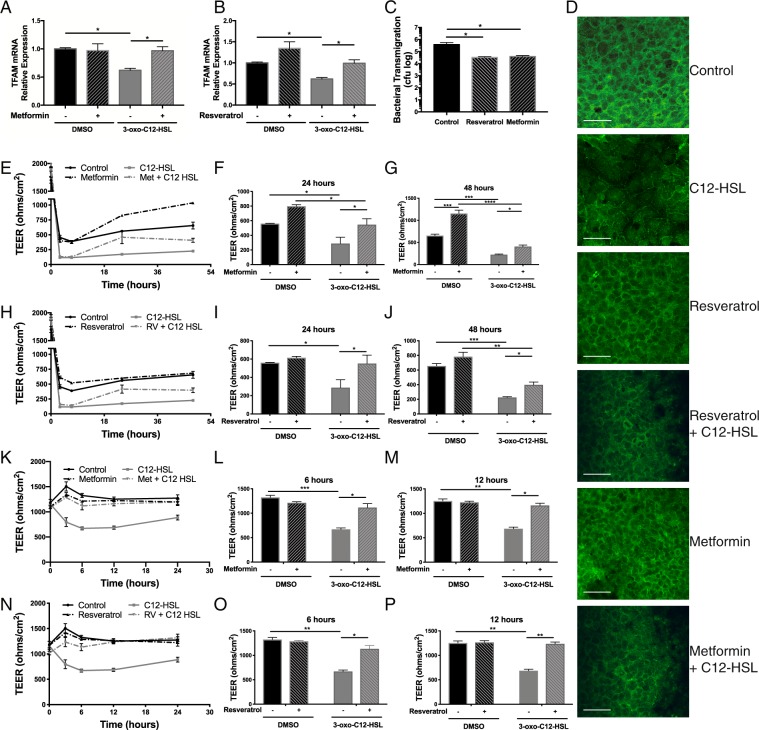
Pre-treatment with metformin or resveratrol partially rescues impairment in mitochondrial biogenesis and restores epithelial barrier integrity following 3-oxo-C12-HSL treatment and prevents bacterial transmigration. (**A**,**B**) BEAS-2B cells were pre-treated with either metformin 1 mM (**A**) or resveratrol 20 μM (**B**) for 24 hours prior to 12-hour treatment with 100 μM 3-oxo-C12-HSL and relative mRNA expression of TFAM was measured by QPCR. The attenuation in TFAM expression caused by 3-oxo-C12-HSL was blocked by prior treatment with metformin or resveratrol. (**C**) Calu-3 cells grown on transwell inserts were pretreated with either vehicle control, metformin 1 mM, or resveratrol 20 μM overnight and then inoculated with PAO1 (MOI 1) in the apical media for 6 hours. The basolateral media was collected, serially diluted, and cultured to quantify cfu. Resveratrol or metformin pretreatment significantly decreased the degree of bacterial transmigration. (**D**) BEAS-2B cells pretreated with either metformin1 mM or resveratrol for 24 hours prior to overnight treatment with 100 μM 3-oxo-C12-HSL were examined by immunofluorescence microscopy staining for the tight junction protein, ZO-1. Treatment with 3-oxo-C12-HSL distorted intercellular distribution of ZO-1. These changes were attenuated in cells pretreated with resveratrol or metformin. 60x magnification, Scale bar = 50 μm. (**E**–**J**) Calu-3 were cells grown on transwell inserts and pre-treated overnight with either vehicle control, metformin 1 mM (**D**–**F**) or resveratrol 20 μM (**G**–**I**). Transepithelial electrical resistance (TEER) was then measured at baseline and at regular intervals following treatment with 100 μM 3-oxo-C12-HSL (**E**,**H**). 3-oxo-C12-HSL significantly reduced TEER within 2 hours and this effect persisted for at least 48 hours. Pre-treatment with metformin lead to restoration of barrier integrity at 24 (**E**) and 48 (**F**) hours despite 3-oxo-C12-HSL treatment. A similar effect was seen with resveratrol at 24 (*H*) and 48 (**I**) hours. (**E**–**J**) Primary NhBEs cultured on transwell inserts were pretreated with either vehicle control, metformin 1 mM (*K-M*), or resveratrol 20 μM (**N**–**P**). TEER was then measured before and at 3, 6, 12, and 24 hours following treatment with 100 μM 3-oxo-C12-HSL. Results are mean ± SEM. *p < 0.05, **p < 0.01, ***p < 0.001, ****p < 0.0001 by one-way ANOVA with Tukey’s multiple comparisons test, n = 5 independent experiments (**A**–**C**) or two-way ANOVA with Tukey’s multiple comparisons test, n = 5 independent experiments (**E**–**J**) n = 6 (**K**–**P**).

Given the beneficial effects of a genetic strategy of PGC-1α overexpression on cellular bioenergetics, we next wanted to investigate whether a pharmacologic strategy targeting enhancement of PGC-1α activity would rescue functional improvements in lung epithelial cells. PGC-1α activity is induced by post-translational modifications including phosphorylation by AMPK and deacetylation by SIRT1^25^. For these studies we utilized resveratrol, a potent activator of SIRT1, and metformin, a canonical activator of AMPK, to determine whether PGC-1α could rescue the functional effects of QS molecules on host epithelial cells. We found that 24-hour pre-treatment with resveratrol (20 μM) or metformin (1 mM) prevented attenuation of TFAM expression by QS molecules in BEAS-2B cells (Fig. 8A,B).

In our experimental model, BEAS-2B cells could not form a tight epithelial barrier (baseline trans-epithelial electrical resistance [TEER] measurements did not exceed 200 Ω x cm^2^). Therefore, we utilized Calu-3 lung epithelial cells that form tight monolayers on transwell supports. In Calu-3 cells inoculated with PAO1 (MOI 1) on the apical chamber for 6 hours, pre-treatment with resveratrol (20 μM) or metformin (1 mM) significantly reduced the transmigration of bacteria across the epithelial barrier (Fig. 8C). Further, 3-oxo-C12-HSL disrupted the distribution of the tight junction-associated protein, Zonula Occludens-1 (ZO-1), as detected by immunofluorescence microscopy. Pretreatment with resveratrol or metformin resulted in preserved tight junction architecture (Fig. 8D). In additional experiments in Calu-3 cells, treatment with the QS molecule, 3-oxo-C12-HSL, caused a significant decrease in TEER that persisted over several days (Fig. 8E,H). However, a 24-hour pre-treatment with resveratrol (20 μM) or metformin (1 mM) restored barrier function 24- and 48-hours following treatment with the QS molecule 3-oxo-C12-HSL (Fig. 8F,G,I,J). In primary human airway cells, pre-treatment with metformin or resveratrol prevented the early (3–12 hour) decline in TEER caused by 3-oxo-C12-HSL and protected barrier integrity up to 24 hours following treatment (Fig. 8K–M,N–P). Collectively, these data for the first time show that pharmacologic approaches to induce PGC-1α may help restore barrier integrity thus improving immune function preventing transmigration of bacteria.

## Discussion

**Figure 9:**
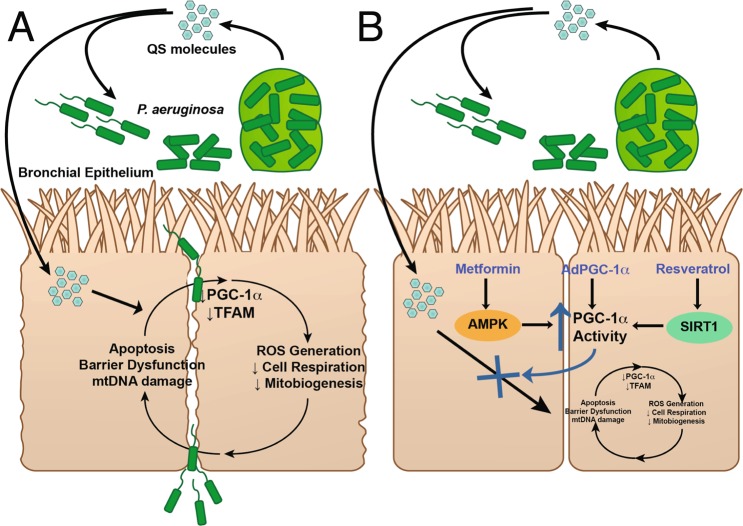
Schematic of the effect of *P*. *aeruginosa* QS molecules on lung epithelial host response. (**A**) QS molecules disrupt mitochondrial bioenergetics, attenuate cellular respiration, induce ROS generation and the apoptosis pathway, repress the PGC-1α-TFAM mitochondrial biogenesis pathway, and trigger loss of barrier integrity. (**B**) Therapy targeting activation of the PGC-1α pathway via genetic or pharmacologic approaches, partially rescues the impairments in mitochondrial respiration, mitochondrial biogenesis, and barrier integrity.

In this study, we have identified a key pathway that is involved in the pathogenesis of lung epithelial injury caused by a virulence factor produced by *P*. *aeruginosa*. 3-oxo-C12-HSL, the principal quorum sensing molecule used by *P*. *aeruginosa*, disrupts mitochondrial morphology, decreases bioenergetic potential, attenuates cellular respiration, enhances ROS generation and subsequent oxidative mtDNA damage, and induces apoptosis in. It also decreases expression of mitochondrial proteins, PGC-1α and TFAM, and decreases mtDNA content, indicative of a reduction in mitochondrial biogenesis (Fig. 9A) in both cell line and primary human lung epithelial cells. Given the central role of PGC-1α as a master regulator of mitochondrial biogenesis, mitochondrial respiration, and anti-oxidant defenses, we hypothesized that activation of the PGC-1α pathway would ameliorate these deleterious effects. Genetic overexpression of PGC-1α does partially rescue markers of mitochondrial biogenesis and cellular respiration. Furthermore, pharmacologic activation of PGC-1α with the agents, metformin or resveratrol, restores epithelial barrier integrity and mitochondrial biogenesis following exposure to 3-oxo-C12-HSL and prevents bacterial transmigration across the epithelial monolayer (Fig. 9B). These data provide a rationale for a novel therapeutic strategy for *P*. *aeruginosa* lung infections.

Mitochondria play a central role in the generation of energy necessary to supply cellular functions. In addition, mitochondria regulate important second messenger cellular signaling pathways including calcium signaling and the induction of the intrinsic apoptosis pathway through cytochrome c release. Given these important functions and perhaps owing to the ancestral origin of mitochondria as a product of bacterial endosymbiosis, it is not surprising that these organelles are frequent targets of bacterial virulence factors^28^. In the lung, mitochondrial function is critical for the proper function of the epithelium including the maintenance of barrier integrity, ciliary function, and the regeneration of epithelial cells following injury^12^. Therefore, mitochondrial dysfunction can result in cellular and tissue dysfunction and promote perpetuation of infection. In addition, damaged mtDNA can act as a danger-associated molecular pattern (DAMP) that can activate TLR9 on nearby immune cells. Further, mitochondrial ROS and mtDNA can activate the NLRP3 inflammasome thereby resulting in increased tissue inflammation and organ injury^29^.

*P*. *aeruginosa* QS molecules, specifically acyl-homoserine lactones, have been shown to affect host epithelial cells in several ways including loss of barrier integrity^30,31^, induction of apoptosis^22,31,32^, and cytosolic calcium release^33,34^. These molecules have also been shown to induce ROS generation in intestinal epithelial cells^32^. We demonstrate for the first time that QS molecules also disrupt mitochondrial morphology, attenuate metabolic potential, induce ROS production and oxidative injury to mtDNA in lung epithelial cells. In addition, 3-oxo-C12-HSL markedly reduces oxidative phosphorylation and ATP production in lung epithelial cells. Interestingly, Tao *et al*. found that 3-oxo-C12-HSL enhanced activity of the respiratory complexes IV and V in intestinal goblet cells^35^. This suggests that there may be cell type- and tissue- specific effects of QS molecules on cellular bioenergetics, which may contribute to the predilection of *P*. *aeruginosa* to cause respiratory infections.

In the face of mitochondrial injury, cells have evolved finely tuned processes designed to maintain tight regulation of mitochondrial quality control. The elimination and replacement of damaged mitochondria are important for the promotion of cell survival. Mitochondrial biogenesis, the process of creating new mitochondria, results from growth and division of existing mitochondria and requires the coordinated activity of PGC-1α and TFAM. We demonstrate for the first time that *P*. *aeruginosa* QS molecules reduce expression of these key regulators in immortalized and primary human lung epithelial cells further supporting the relevance of this finding to clinical infections in humans. In addition, decreased levels of mitochondrial DNA and expression of the mitochondrial marker, VDAC1, demonstrates a reduction in mitochondrial biogenesis. This is a novel finding not previously reported in any cell types. There is scant research highlighting the effect of bacterial pathogens on host mitochondrial biogenesis. One previous study in a murine model of *Staphylococcus aureus* pneumonia found that mitochondrial biogenesis was upregulated during lung repair in the distal lung epithelium following antibiotic treatment^14^. Others have found that mitochondrial biogenesis is attenuated early in other sepsis models with recovery over time^36,37^. In addition, dysregulation of mitochondrial biogenesis potentiated the degree of liver injury in an experimental model of sepsis^36^. Finally, a recent study by Cui *et al*. demonstrated that PGC-1α is downregulated in mouse alveolar epithelial cells exposed to LPS and conditional deletion of PGC-1α in these cells aggravated a mouse model of acute lung injury^38^. These studies support the idea that PGC-1α, through its promotion of cellular bioenergetics and fatty acid oxidation serves a protective role in the homeostatic host response to inflammatory stimuli. Given the importance of proper mitochondrial function in maintaining epithelial barrier integrity and host response^11–13^, disruption of mitochondrial function and the mitochondrial biogenesis repair process may reflect an additional virulence mechanism employed by *P*. *aeruginosa*.

We hypothesized that a strategy targeting activation of PGC-1α to enhance cellular respiration, mitochondrial biogenesis, and anti-oxidant defenses could protect the epithelial host response to *P*. *aeruginosa* infection. Such a strategy has had efficacy in models of other tissue injury including pulmonary fibrosis^16^, hepatic ischemia-reperfusion injury^39^, kidney injury^40^, and aging-related degeneration in the mouse lung^41^. Using an adenovirus overexpression strategy, we found that upregulation of PGC-1α attenuated the reduction in mitochondrial respiration induced by 3-oxo-C12-HSL. In addition, we employed two widely available chemicals, resveratrol and metformin, that act through complementary mechanisms to activate PGC-1α via post-translational modifications. These molecules resulted in more rapid recovery of barrier integrity following QS molecule treatment. This provides preliminary evidence that enhancement of PGC-1α activity represents a novel therapeutic strategy for *P*. *aeruginosa* infection.

A limitation of this paper is that we did not investigate other homeostatic mitochondrial quality control processes that work alongside mitochondrial biogenesis, specifically mitophagy. Ongoing studies are necessary to delineate whether mitophagy is disrupted in host lung epithelial cells by QS molecules. Another limitation derives from the fact that resveratrol and metformin exert many pleotropic effects through their effects on AMPK or SIRT1 in addition to activation of PGC-1α. Further investigations are needed to clarify the exact mechanisms by which metformin and resveratrol restore barrier integrity. In addition, a limitation of this manuscript is the lack of animal studies examining the effect of bacterial infection on *in vivo* epithelial mitochondrial function. These and other ongoing studies in our laboratory will further define the precise role of PGC-1α in mitochondrial host response to invading pathogens.

In conclusion, these studies provide a new paradigm and implications for the role of mitochondria in the epithelial cell host response to *P*. *aeruginosa* infections. Our data suggest that novel molecular approaches to rescue mitochondrial function may enhance immune responses to pathogens such as *P*. *aeruginosa*. Since drugs such as metformin and resveratrol that can activate PGC-1α are already in clinical use, these results are translatable to patients with *P*. *aeruginosa* infections and other pathogens that evade immune defenses by similar mechanisms.

## Methods

### Cell line models

BEAS-2B bronchial epithelial cells (ATCC, Rockville, MD) were maintained with complete BEGM media (Lonza, Basel, Switzerland) supplemented with 10% FBS and plated on dishes coated with collagen-fibronectin-BSA mixture. Calu-3 cells (ATCC) were maintained in EMEM media supplemented with 10% FBS plated on 3 μM polyester membrane transwell supports (Corning, Corning, NY). After plating cells, epithelial tight junctions were allowed to mature over 7–10 days until the TER was over 1000 Ω x cm^2^. Cells were treated with 3-oxo-C12-HSL (Sigma-Aldrich, St. Louis, MO), trans-resveratrol (Cayman Chemical, Ann Arbor, MI), or metformin (Cayman Chemical), or co-cultured with the *P*. *aeruginosa* strain, PAO1, (MOI of 1 or 30), for 6 or 16 hours. Primary NhBE cells were grown in conditional reprogrammed cell (CRC) culture medium as described in Liu *et al*.^42^, on human type IV placental collagen-coated 12-mm Costar Transwell supports (Corning) with slight modifications, by the CF@LANTA RDP Experimental Models Core at Emory University as described previously^43^.

### Bacterial stocks

Stock of *P*. *aeruginosa* strain PAO1 was prepared as previously described^44^. Bacteria were used at a multiplicity of infection (MOI) of 30 for 6-hour and MOI of 1 for 16-hour *in vitro* experiments unless otherwise stated. Cultures were adjusted to an OD600 (optical density) of 0.2 (∼1.86 × 10^9^ colony-forming units/ml)^45^.

### Transmission electron microscopy (TEM)

Monolayer cells were fixed with 2.5% glutaraldehyde in 0.1 M cacodylate buffer (pH 7.4). Cells were then rinsed with 0.1 M cacodylate buffer (pH 7.4) twice before post-fixation in 1% osmium tetroxide for 1 hour. After additional buffer rinses, cells were dehydrated through an ethanol series to 100% ethanol. Cells were infiltrated with a mixture of 100% ethanol and Eponate 12 resin (Ted Pella Inc., Redding, CA), and then pure Eponate 12 resin overnight. Cells were embedded in multiwall plate and then placed in a 60 °C oven for polymerization. Ultrathin sections were cut on a Leica UltraCut microtome at 70–80 nm and placed on 200 mesh copper grids. Sections were then stained with 5% uranyl acetate for 15 minutes followed by 2% lead citrate for 15 minutes. Cells were imaged with a JEOL JEM-1400 transmission electron microscope (Tokyo, Japan) equipped with a Gatan US1000 CCD camera (Pleasanton, CA) by a blinded observer. At least 10 high-powered images (15,000x, 18 μm^2^) were examined and various variables were quantified with software analysis using Image J software (National Institutes of Health, Bethesda MD).

### Mitochondrial bioenergetics

The Seahorse XF analyzer Cell Energy Phenotype assay (Agilent Technologies, Santa Clara, CA) was performed on BEAS-2B cells treated for 6 hours with C12-HSL (100 μM). OCR and ECAR were measured before and after treatment of cells with mitochondrial toxins: the ATP synthase inhibitor, oligomycin (1 μM), and the uncoupling agent, Carbonyl cyanide-4-phenylhydrazone (FCCP, 0.25 μM), to determine baseline and stressed measures of oxidative phosphorylation and glycolysis, respectively. The Seahorse XF analyzer Mito Stress Test assay (Agilent Technologies) was also performed on BEAS-2B cells treated for 6 hours with C12-HSL (50, 100, and 200 μM). Oxygen consumption rate is plotted over time in response to a series of injections with inhibitors of the electron transport chain: oligomycin (1 μM), FCCP (0.25 μM), and then rotenone and antimycin A (0.75 μM). OCR and ECAR values were normalized to cell number.

### ATP concentration

The ATP Determination Kit (Molecular Probes, Eugene, OR), a bioluminescence assay for quantitative determination of ATP, was used to assess steady state levels of ATP in cell lysates. 3 μg cell lysate samples were loaded in triplicate into a 96 well plate luminometer and the assay was conducted per manufacturer instructions. An ATP standard curve was generated. Luminescence of samples and standards was measured in triplicate per the manufacturer instructions and the quantity of ATP was calculated based on the standard curve.

### ROS detection

Cells were detached with warm accutase, then collected by centrifugation, and re-suspended in media. MitoSOX™ Red (Invitrogen) was added at a final concentration of 5 mM for 20 mins at 37 °C. The cells were washed once in warm PBS and immediately analyzed by FACS analysis. Cells were acquired using BD FACSAria II and analyzed using FACSDiva software. The level of intracellular ROS corresponded to an increase in fluorescence. Cells expressing fluorescence above a defined threshold were considered positive and were evaluated based on the population defined by double discrimination gating and the unstained and untreated controls.

### Quantitative-RT-PCR

RNA was isolated using RNeasy kit (Qiagen). 1 μg of RNA was used to synthesize cDNA using SuperScript II RT (Invitrogen). Quantitative real time PCR was performed using SYBR green probes (Applied Biosystems) normalized to the internal control GAPDH using the ΔΔCt method. Primer sequences for SYBR green probes are the same as used previously^46^: PGC-1α (Forward 5′-ACTGAGCTACCCTTGGGATG-3′, Reverse 5′-TAAGGATTTGGGTGGTGACA-3′); TFAM (Forward 5′-GAACAACTACCCATATTTAAAGCTCA-3′, Reverse 5′-GAATCAGGAAGTTCCCTCCA-3′); GAPDH (Forward: 5′-GCCCAATACGACCAAATCC-3′, Reverse: 5′-AGCCACATCGCTCAGACAC-3′); VDAC1 (Forward 5′-CAGGCTCCTGTGTCTGCTG-3′, Reverse 5′-GAAGACATCCCTGGCAGATT-3′).

### Western blotting

Protein extraction, electrophoresis, and gel transfer to nitrocellulose membranes were performed as previously described^43^. The following primary antibodies were used: rabbit anti-PGC-1α (2G6, 1:400), rabbit anti-TFAM (D5C8, 1:500), rabbit anti-PARP (#9542, 1:500), rabbit anti-cleaved caspase-9 (Asp391/18C8, 1:500), mouse anti-caspase-8 (#9746, 1:500), rabbit anti-cleaved caspase-9 (Asp 315, 1:500), rabbit anti-caspase-9 (#9502, 1:500) from Cell Signaling Technology (Danvers, MA), and mouse anti-GAPDH (1:1000, Santa Cruz Biotechnology, Dallas, TX). For PGC-1α and TFAM immunoblots, membranes were incubated with HRP-conjugated goat anti-rabbit IgG (1:1000), developed with West Dura substrate (Thermo Fisher Scientific), and imaged on Amersham Hyperfilm™ ECL film (GE Healthcare Bio-Sciences, Pittsburgh, PA). For other immunoblots, blots were incubated with IRDye® 800CW conjugated polyclonal goat anti-mouse IgG (1:10,000) or IRDye® 680RD conjugated polyclonal goat anti-rabbit IgG (1:5,000) from LI-COR biosciences (Lincoln, NE) and imaged using the Odyssey Infrared Imaging System (LI-COR Biosciences). When described, blots were stripped with Restore™ stripping buffer (Thermo Fisher Scientific) when probing with alternative antibodie.

### Mitochondrial DNA content

Relative mitochondrial DNA content was determined as described previously^47,48^. Briefly, DNA was isolated from samples using DNeasy kit (Qiagen) per manufacturer instructions. DNA was quantified using NanoDrop spectrophotometry (Thermo Fisher Scientific, Washington, DE). 40 ng DNA of each sample was amplified with mtDNA tRNA^Leu(UUR)^ and nuclear β-2-microglobulin primer pairs (tRNA^Leu(UUR)^ Forward: 5′-CACCCAAGAACAGGGTTTGT-3′, Reverse: 5′-TGGCCATGGGTATGTTGTTA-3′; β-2-microglobulin Forward: 5′-TGCTGTCTCCATGTTTGATGTTGTATCT-3′; Reverse: 5′-TCTCTGCTCCCCACCTCTAAGT-3′). The mtDNA tRNA^Leu(UUR)^ gene region was selected because it is rarely deleted or duplicated and contains only a few rare SNPs. Relative mtDNA content was normalized to nuclear DNA using the ΔΔC_T_ method.

### Caspase activity assay

Caspase-3/CPP32 colorimetric assay kit (BioVision, Mountain View, CA) was used to determine caspase activity per manufacturer’s instructions as previously described^49^. Cells were treated and collected with cell lysis buffer. An equal amount of protein was loaded per assay. Absorbance was measured at 405 nm on a microplate reader. Caspase activity is expressed as fold change in comparison to untreated control cells.

### Cytochrome C releasing assay

The water soluble mitochondrial inner membrane protein, cytochrome c was detected by solid phase sandwich enzyme-linked immune-sorbent assay (ELISA, Invitrogen, Camarillo, CA) to detect an early component required for intrinsic apoptosis initiation per manufacturer instructions as previously described^50^. Samples (3 μg protein per well) and standard containing cytochrome c were loaded into wells coated with a monoclonal antibody specific for cytochrome c. Next, a biotinylated secondary antibody specific for cytochrome c is added. Following addition of streptavidin peroxidase enzyme, a substrate solution was added to the wells to produce color detected at 450 nm on a microplate reader. A standard curve was generated and the concentration of each sample was then calculated.

### Mitochondrial DNA damage

Mitochondrial DNA integrity was assessed as described previously^51–53^. Briefly, genomic DNA was extracted using DNeasy kit (Qiagen) and quantitative PCR was used to measure the relative content of the copy number of short mtDNA-79 bp fragments (indicative of damaged mtDNA) to the copy number of the long mtDNA-230 fragments (indicative of undamaged mtDNA) of the 16S-RNA gene. The sequence for the forward primer (both, mtDNA79 and mtDNA230) was 5′-CAGCCGCTATTAAAGGTTCG-3′, and the reverse primers were 5′-CCTGGATTACTCCGGTCTGA-3′ (mtDNA79) and 5′-GGGCTCTGCCATCTTAACAA-3′ (mtDNA230).

### Adenovirus overexpression of PGC-1α

An adenovirus expressing PGC-1α, AdPGC-1α (a gift from Dr. Russ Price, East Carolina University) was used as described previously^46^. Briefly, BEAS-2B cells were transduced with AdPGC-1α (MOI 25) in BEGM media containing 2% FBS. Media was changed to full 10% FBS media 6 hours following transduction. RNA was isolated 48 hours following transduction. Protein was isolated and Seahorse bioenergetics assays were performed 72 hours following transduction.

### TEER

Calu-3 cells were grown on 3 μM polyester membrane Costar Transwell® permeable supports (Corning) and tight junctions were matured after a period of 7–10 days. Alternatively, differentiated primary NHBes were grown on type IV placental collagen-coated 6.5-mm Costar Transwell supports. TER was measured using an Ohmmeter (World Precision Instruments, Sarasota, FL) with chopstick electrodes as previously described^43^. Cells were pretreated with resveratrol or metformin for 24 hours prior to treatment with 100 μM 3-oxo-C12-HSL. TER was measured at baseline and at serial intervals following treatment.

### Bacterial transmigration assay

Calu-3 grown on Transwell® permeable supports as above were pretreated with resveratrol or metformin for 18 h. Bacterial suspension (MOI of 1) was added apically. After 6 hours, the apical and basolateral media were collected separately. A sample of the basolateral medium was serially diluted, cultured on LB agar, and incubated at 37 °C overnight. Colony-forming units were recorded (accurate range, ≥30 ≤ 300), as described previously^43^.

### Immunofluorescence

BEAS-2B cells were plated on 8-well chamber slides (Thermo Fisher Scientific) coated with collagen, fibronectin, and BSA, as described above. Immunofluorescence was performed on cells as follows: post-treatment chamber slides were washed in PBS twice and fixed with 2% paraformaldehyde for 15 minutes. Cells were then permeabilized with 0.1% Triton-X-100 and blocked with PBS (with calcium and magnesium) that contained 0.1% Triton-X-100 and 2% goat serum (Sigma-Aldrich). Cells were incubated with rabbit polyclonal anti-ZO-1 antibody (Thermo Fisher Scientific, #617300) overnight at 4 °C and then incubated with fluorescent-tagged (Alexa Fluor® 488) secondary goat anti-rabbit IgG (Thermo Fisher Scientific) for 1 hour at room temperature. Slides were mounted with ProLong Gold mounting media (Thermo Fisher Scientific). Images were obtained using Olympus Fluoview FV1000 confocal microscope.

### Statistical analysis

All experiments were repeated at least 3 times. Data are presented as mean ± SEM. Detailed information regarding statistical tests used is included in figure legends. All statistical analyses were performed by using GraphPad Prism (GraphPad Software, La Jolla, CA, USA).

## Supplementary information

Supplementary Info
