# Supplementary material for: Pseudomonas aeruginosa Induced Host Epithelial Cell Mitochondrial Dysfunction
Source: Sci Rep. 2019 Aug 15;9:11929. doi: 10.1038/s41598-019-47457-1 (PMC6695387; doi:10.1038/s41598-019-47457-1)
Supplement: Supplementary file 1 — Supplementary Info [file 41598_2019_47457_MOESM1_ESM.pdf]

Supplementary Information

Pseudomonas aeruginosa Induced Host Epithelial Cell Mitochondrial Dysfunction

Nicholas M. Maurice[1,2], Brahmchetna Bedi[1,2], Zhihong Yuan[1,2], Joanna B. Goldberg[3,4], Michael Koval[1,5], C. Michael Hart[1,2], Ruxana T. Sadikot[1,2*]

[1] Department of Medicine, Division of Pulmonary, Allergy, Critical Care, and Sleep Medicine, Emory University School of Medicine, Atlanta, GA, 30322; [2] Atlanta Veterans Affairs Health Care System, Decatur, GA, 30033; [3] Department of Pediatrics, Division of Pulmonology, Allergy/Immunology, Cystic Fibrosis, and Sleep, Emory University, Atlanta, GA, 30322; [4] Children's Healthcare of Atlanta, Center for CF and Airways Disease Research Atlanta, GA, USA; [5] Department of Cell Biology, Emory University School of Medicine, Atlanta, GA 30322.

* Corresponding Author: Ruxana T. Sadikot, M.D., M.R.C.P., Emory University School of Medicine, Department of Medicine, Division of Pulmonary, Allergy, Critical Care and Sleep Medicine, Atlanta Veterans Affairs Health Care System, 1670 Clairmont Road, Decatur, GA 30033; ruxana.sadikot@emory.edu; Tel (404) 321-6111; Fax. (404) 728-7733.

Supplementary Figure 1

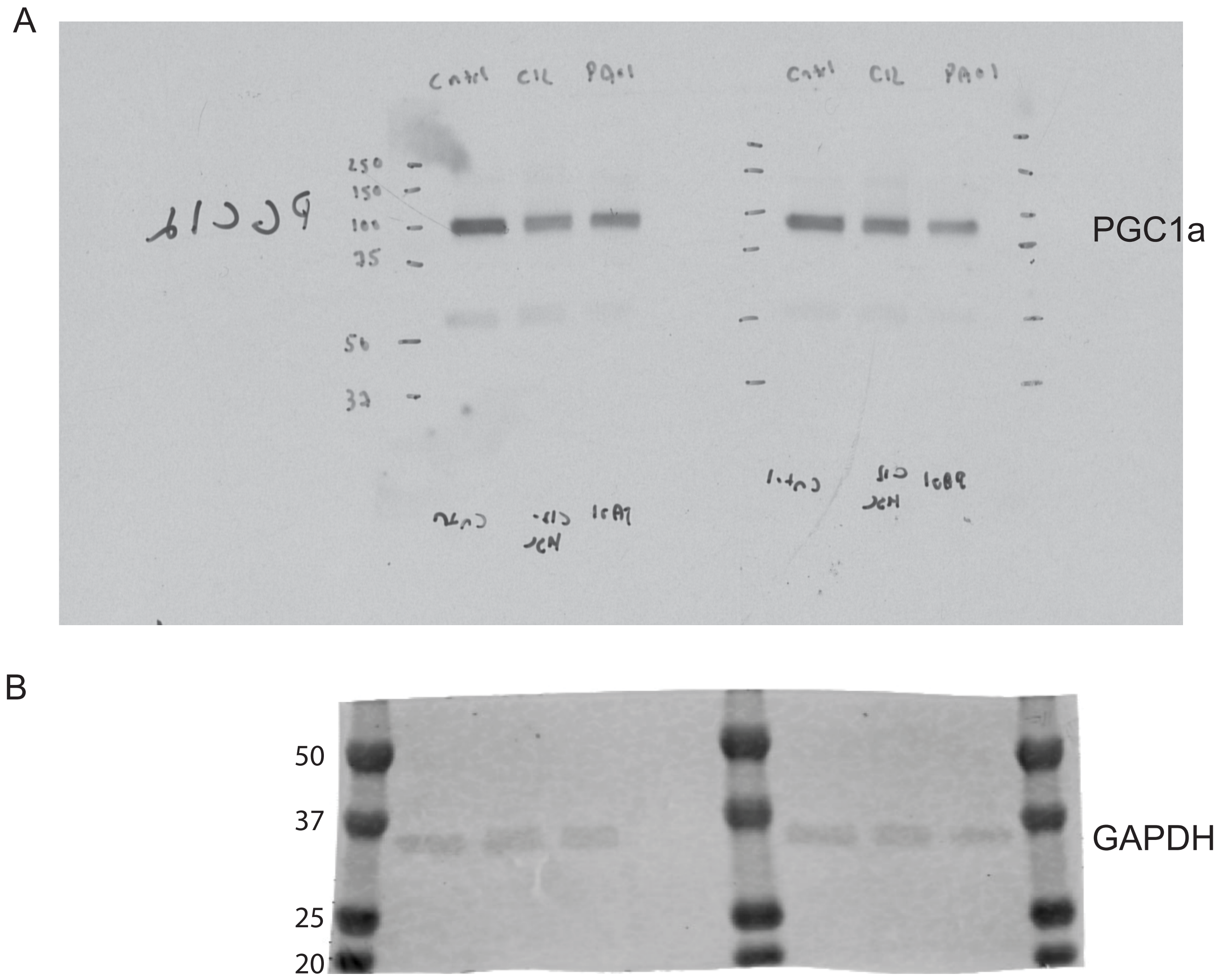

Supplementary Figure 1. 3-oxo-C12-HSL and PAO1 decrease protein expression of PGC-1α.
A, Full immmunoblot for PGC-1α. The bands on the left correspond to the cropped blot used in Fig. 4B. The image was obtained using chemiluminescent substrate and imaged using film. *B*, Full immunoblot for GAPDH loading control. After PGC-1α immunoblotting, the membrane was stripped and cut at 50 kD. The lower portion of the membrane was incubated with GAPDH antibody and IRDye 800CW conjugated polyclonal goat anti-mouse IgG and imaged using the Odyssey Infrared Imaging System.

!

Supplementary Figure 2

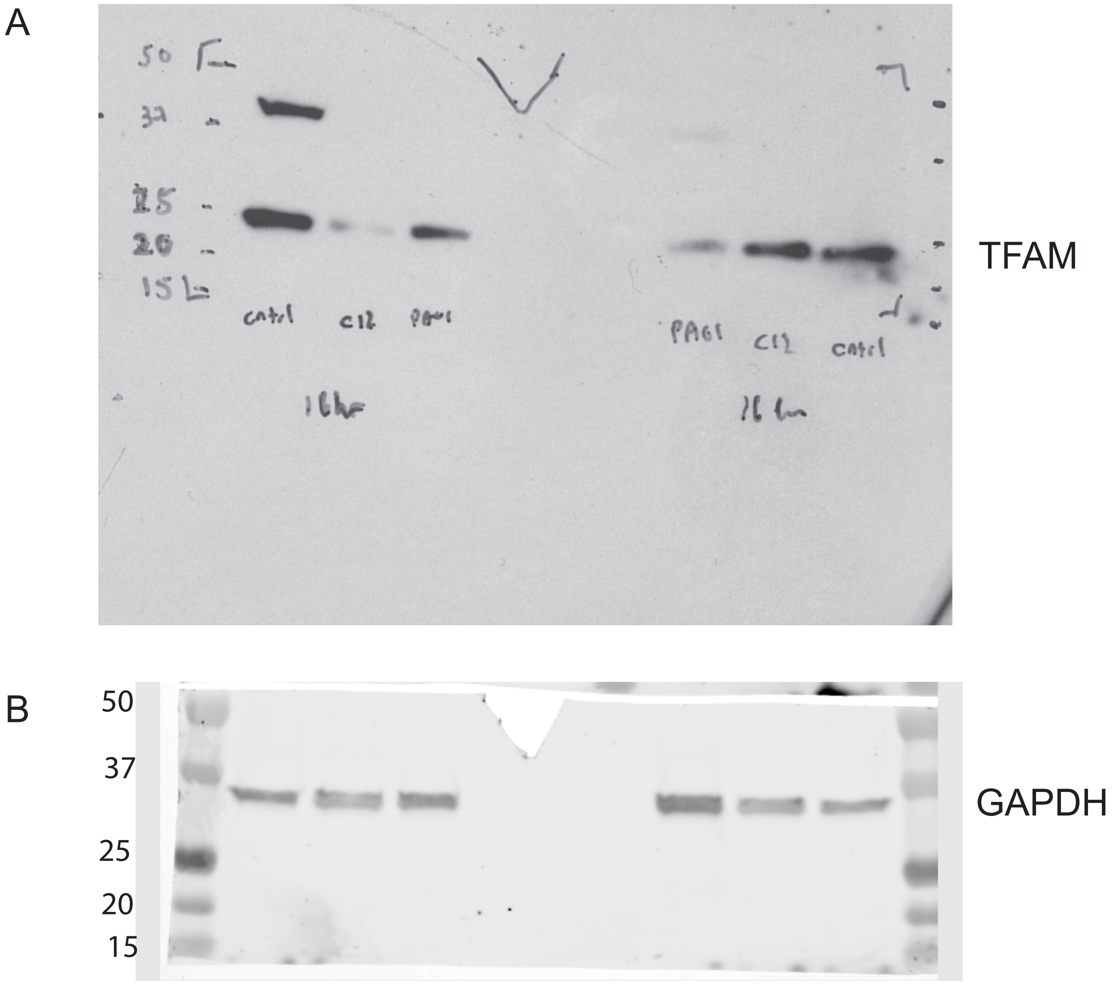

Supplementary Figure 2. 3-oxo-C12-HSL and PAO1 decrease protein expression of TFAM.

A, Full immmunoblot for TFAM. The membrane was cut at 50 kD after transfer. The lower portion of the blot was subsequently used. The bands on the left correspond to the cropped blot used in Fig. 4F. The image was obtained using chemiluminescent substrate and imaged using film. . *B*, Full immunoblot for GAPDH loading control. The image was obtained using the Odyssey Infrared Imaging System..

Supplementary Figure 3

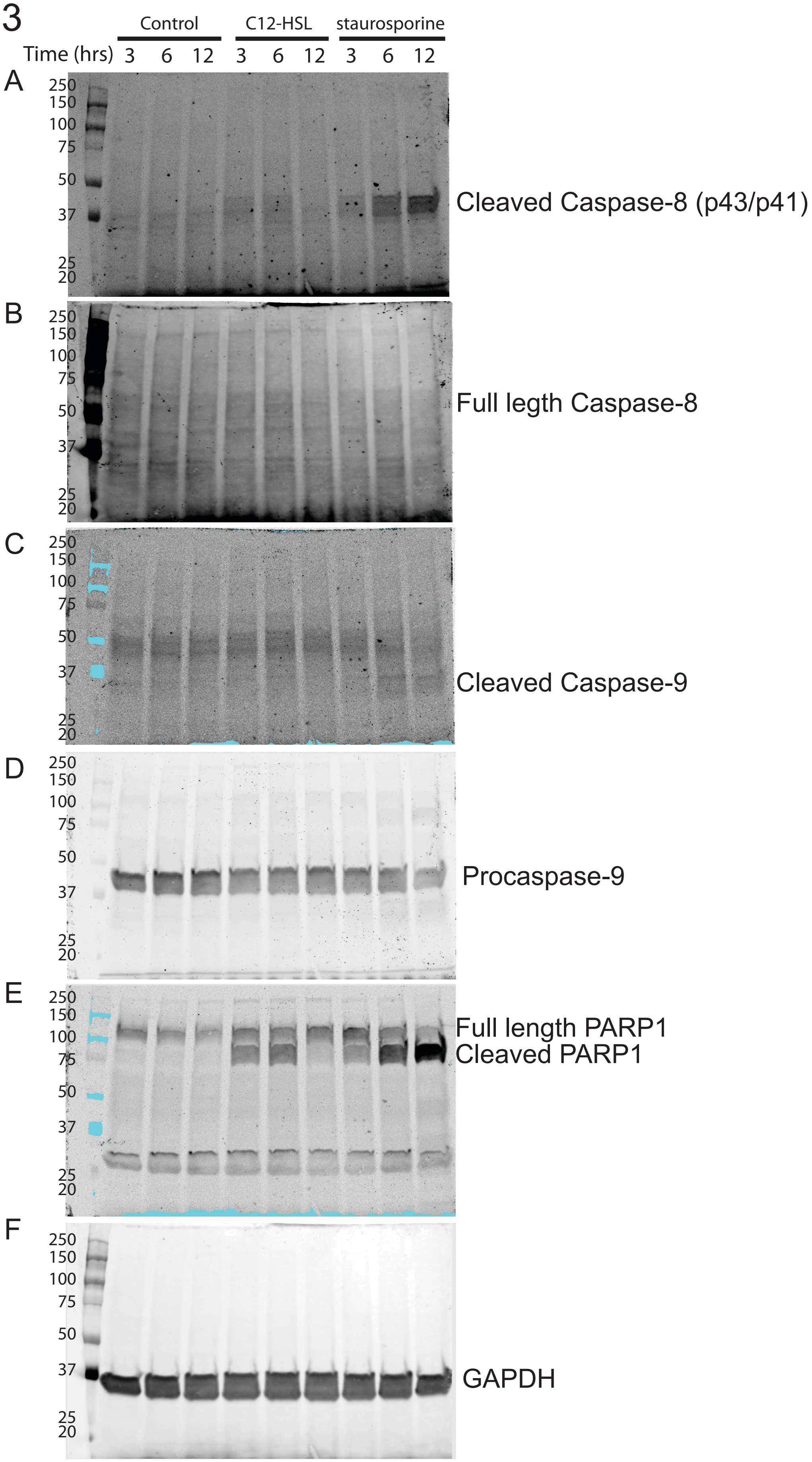

Supplementary Figure 3. 3-oxo-C12-HSL induces apoptosis in bronchial epithelial cells.
Full Immunoblots for cleaved caspase-8 (*A),* full-length caspase-8 (*B*), cleaved caspase-9 (*C*), procaspase-9 (*D*), PARP1 (*E*), and GAPDH (*F*). Aftering probing with primary antibody, membranes were incubated with IRDye 800CW conjugated polyclonal goat anti-mouse or anti-rabbit IgG and imaged using the Odyssey Infrared Imaging System. After visualization, membranes were stripped with Restore stripping buffer and then reprobed with antibody below and the process was repeated. Staurosporine-treated cells were used as a positive control. *A,B,E,* and *F* correspond to Figure 6B.

Supplementary Figure 4

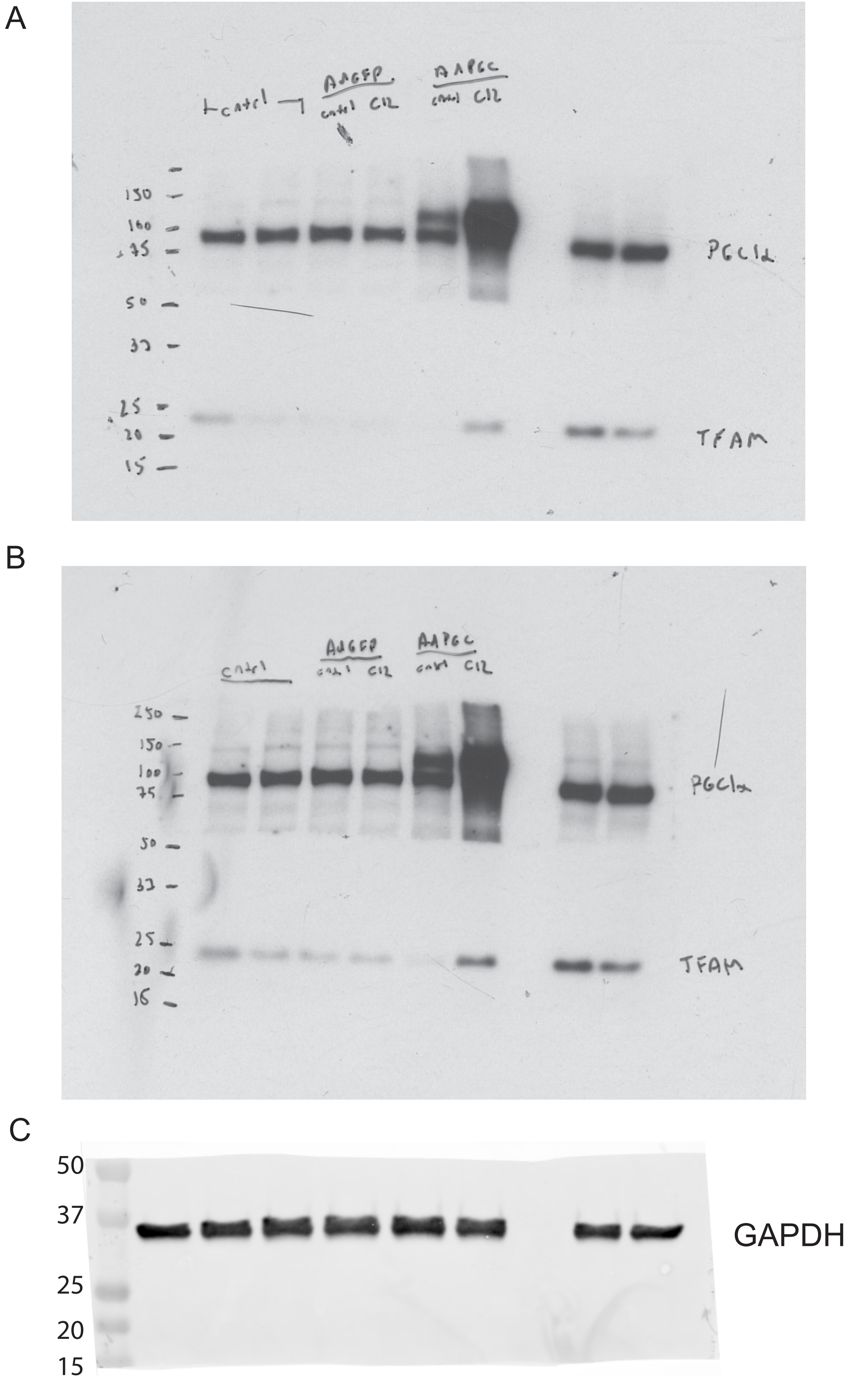

Supplementary Figure 4. Adenoviral overexpression of PGC-1α prevents the attenuation in the expression of PGC-1α and TFAM caused by 3-oxo-C12-HSL. (*A*) Full immunoblot for PGC-1α (top) and TFAM (bottom) using a short exposure time. The membrane was cut at 50 kD after transfer. The upper portion of the membrane was used for PGC-1α immunoblotting and the bottom portion was used for TFAM and GAPDH immunoblotting. (*B*) Full immunoblot for PGC-1α (top) and TFAM (bottom) using a longer exposure time. The images in A and B were obtained on film. (*C*) Full immunoblot for GAPDH loading control obtained using the Odyssey Infrared Imaging System.
